# Right ventricular to pulmonary arterial coupling in patients with repaired tetralogy of Fallot: a case series

**DOI:** 10.1093/ehjcr/ytad583

**Published:** 2023-12-01

**Authors:** Renée S Joosen, Johannes M P J Breur, Jeroen N Wessels, Gregor J Krings, Michiel Voskuil, Frances S de Man, Marielle C van de Veerdonk

**Affiliations:** Department of Pediatric Cardiology, University Medical Center Utrecht, Utrecht, The Netherlands; Department of Pediatric Cardiology, University Medical Center Utrecht, Utrecht, The Netherlands; PHEniX laboratory, Department of Pulmonary Medicine, Amsterdam UMC location Vrije Universiteit Amsterdam, Amsterdam, The Netherlands; Pulmonary Hypertension and Thrombosis, Amsterdam Cardiovascular Sciences, Amsterdam, The Netherlands; Department of Pediatric Cardiology, University Medical Center Utrecht, Utrecht, The Netherlands; Department of Cardiology, University Medical Center Utrecht, Utrecht, The Netherlands; PHEniX laboratory, Department of Pulmonary Medicine, Amsterdam UMC location Vrije Universiteit Amsterdam, Amsterdam, The Netherlands; Pulmonary Hypertension and Thrombosis, Amsterdam Cardiovascular Sciences, Amsterdam, The Netherlands; Cardiovascular Sciences, Heart Failure and Arrhythmias, Amsterdam UMC, location University of Amsterdam, Meibergdreef 9, Amsterdam 1105 AZ, The Netherlands

**Keywords:** Case series, Tetralogy of Fallot, Right ventricular dysfunction, Pulmonary pressures, Pulmonary stenosis, Cardiac magnetic resonance imaging

## Abstract

**Background:**

In repaired tetralogy of Fallot (ToF) patients with residual right ventricular (RV) outflow tract obstructions (RVOTO), risk stratification and timing of re-interventions are based on RVOTO gradients. However, this might be insufficient to prevent RV dysfunction. Instead, assessment of RV to pulmonary arterial (RV-PA) coupling allows integrated assessment of RV function in relationship to its afterload and could be of additional value in clinical decision-making.

**Case summary:**

Two patients with repaired ToF and residual RVOTO without pulmonary regurgitation underwent right heart catheterization (RHC) and cardiac magnetic resonance imaging. We determined RV end-systolic elastance (Ees), arterial elastance (Ea) and RV-PA coupling (Ees/Ea) using single-beat RV pressure–volume analysis. Patient 1 was asymptomatic despite severely increased RV pressures and a left pulmonary artery (LPA) stenosis (invasive gradient 20 mmHg). Right ventricular volumes and function were preserved. The Ea and Ees were increased but RV-PA coupling was relatively maintained. Of interest, RV end-diastolic pressure and RV diastolic stiffness were increased. After LPA plasty, RV function was preserved during long-term follow-up. Patient 2 was symptomatic despite mildly elevated RV pressures and a supravalvular RV-PA conduit stenosis (invasive gradient 30 mmHg). The RV showed severe RV dilatation and dysfunction. The Ea was increased but Ees was decreased leading to RV-PA uncoupling. Despite balloon angioplasty, RV function was unchanged during long-term follow-up.

**Discussion:**

Development of RV dysfunction might be insufficiently predicted by RVOTO severity in patients with repaired ToF. Assessment of RV remodelling and function in relationship to its afterload might help to optimize risk stratification.

**Learning points**

- Right ventricular (RV) pressures do not always inform us properly about the extent of RV adaptation and RV function.
- This case series is the first to perform single-beat RV pressure–volume analysis in patients with ToF and provides new insights that linking indexes of RV function to its afterload might be of additional value for risk stratification, monitoring, and optimizing timing of re-interventions in patients with repaired ToF.

## Introduction

**Figure 1:**
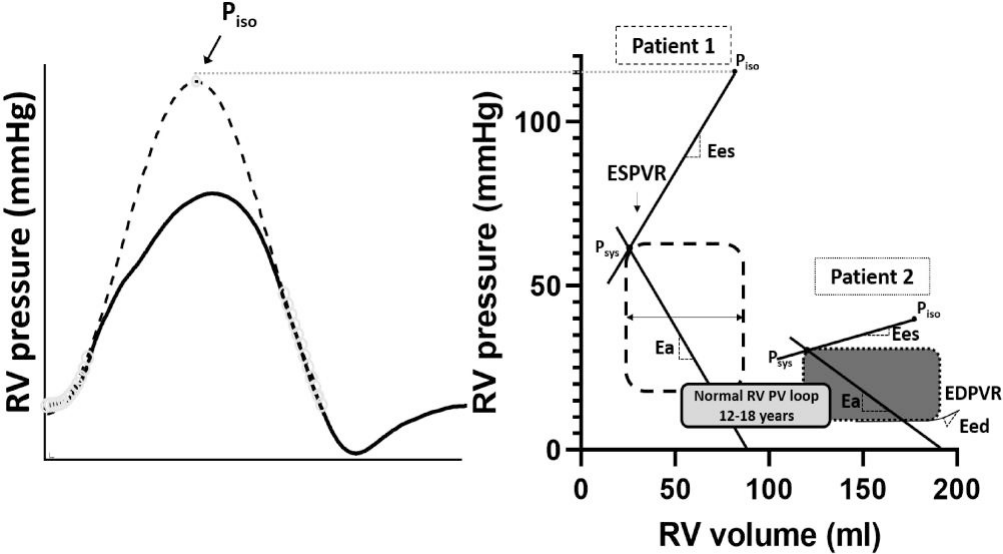
Pressure–volume loops using the single-beat method from Brimioulle et al.^7^*Figure 1* shows the pressure–volume loops of Case 1, Case 2, and a normal RV in the age group 12–18 years. Right ventricular to pulmonary arterial (RV-PA) coupling was calculated as the ratio of the Ees and the arterial Ea and represents the efficiency of energy transfer from the RV to the pulmonary vasculature (Ees/Ea normal RV = ≥1, Ees/Ea Patient 1 = 0.8, Ees/Ea Patient 2 = 0.2). Ees, which is considered a load-independent measure of ventricular contractility, was calculated as the slope of the ESPVR using (RV maximal isovolumetric pressure − RV systolic pressure)/stroke volume (Ees normal RV = ≥0.5, Ees Patient 1 = 0.9, Ees Patient 2 = 0.1). The maximal isovolumetric pressure of the RV was computed by sine wave extrapolation using RV pressure values recorded before maximal first derivative of pressure development over time (dP/dt) and after minimal dP/dt.^7^ Ea, which is mainly a reflection of pulmonary vascular resistance, was calculated as RV afterload using RV systolic pressure/stroke volume (Ea normal RV = ≤0.31, Ea Patient 1 = 1.2, Ea Patient 2 = 0.5).^9^ In addition, diastolic stiffness was assessed using RV Eed. Eed was calculated as the slope of the EDPVR at end-diastole (Eed normal RV = ≤0.24, Ea Patient 1 = 0.9, Ea Patient 2 = 0.2).^10^ EDPVR, end-diastolic pressure–volume relationship; Eed, end-diastolic elastance; Ees, end-systolic elastance; Ea, arterial elastance; Ees/Ea, RV-PA coupling; ESPVR, end-systolic pressure–volume relationship; Piso, maximal isovolumetric pressure of the RV; Psys, systolic pressure of the RV; PV, pressure–volume; RV, right ventricle.

Tetralogy of Fallot (ToF) is the most common cyanotic congenital heart disease. Although survival has improved, patients suffer from residual pulmonary regurgitation (PR) and right ventricular (RV) outflow tract obstruction (RVOTO) leading to RV dysfunction.^1^ Right ventricular dysfunction is independently associated with reduced survival, and therefore, maintenance of RV function is of great importance.^2^ Timing of re-interventions for the relief of RVOTO remains challenging. According to the guidelines, decision-making should be guided by RVOTO gradients.^3,4^ However, a recent study found no association between peak RVOT gradient on echocardiography and the severity of RV dysfunction.^5^ Therefore, integrated assessment of RV remodelling and function in relationship to its afterload could be of additional value in clinical decision-making. The interaction between the RV and pulmonary arterial load can be assessed using pressure–volume loops. Within this framework, RV end-systolic elastance (Ees), which is considered a load-independent measure of ventricular contractility, can be calculated as the slope of the end-systolic pressure–volume relationship (ESPVR) using (RV maximal isovolumetric pressure − RV systolic pressure)/stroke volume. The multi-beat RV pressure–volume analysis, in which multiple pressure volume loops during changing preload conditions are being used to calculate the slope of the ESPVR, is the gold standard to assess Ees.^6^ However, due to the preload alterations, this is often not feasible in patients and the single-beat RV pressure–volume analysis from Brimioulle et al.^7^ can serve as an applicable alternative.^7^ During this single-beat RV pressure–volume analysis, the slope of the ESPVR is being assessed using the maximal isovolumetric pressure of the RV, which can be extrapolated from RV pressure curves (*Figure 1*).^8^

In addition to Ees, the pulmonary arterial elastance (Ea), which is mainly a reflection of pulmonary vascular resistance, can be calculated as RV afterload using RV systolic pressure/stroke volume.^9^ The RV to pulmonary arterial (RV-PA) ‘coupling’ is calculated as the ratio of Ees/Ea and represents the efficiency of mechanical energy transfer from the RV to the pulmonary vasculature. In addition, diastolic stiffness can be assessed using RV end-diastolic elastance (Eed). End-diastolic elastance can be calculated as the slope of the end-diastolic pressure–volume relationship (EDPVR) at end-diastole.^10^ Here, we present two cases with repaired ToF and a supravalvular RVOTO who underwent right heart catheterization (RHC) and cardiac magnetic resonance (CMR) imaging. In addition, single-beat RV pressure–volume analysis was performed.

## Summary figure

**Timeline clinical course of two repaired ToF patients with RV pressure overload**

**Figure:**
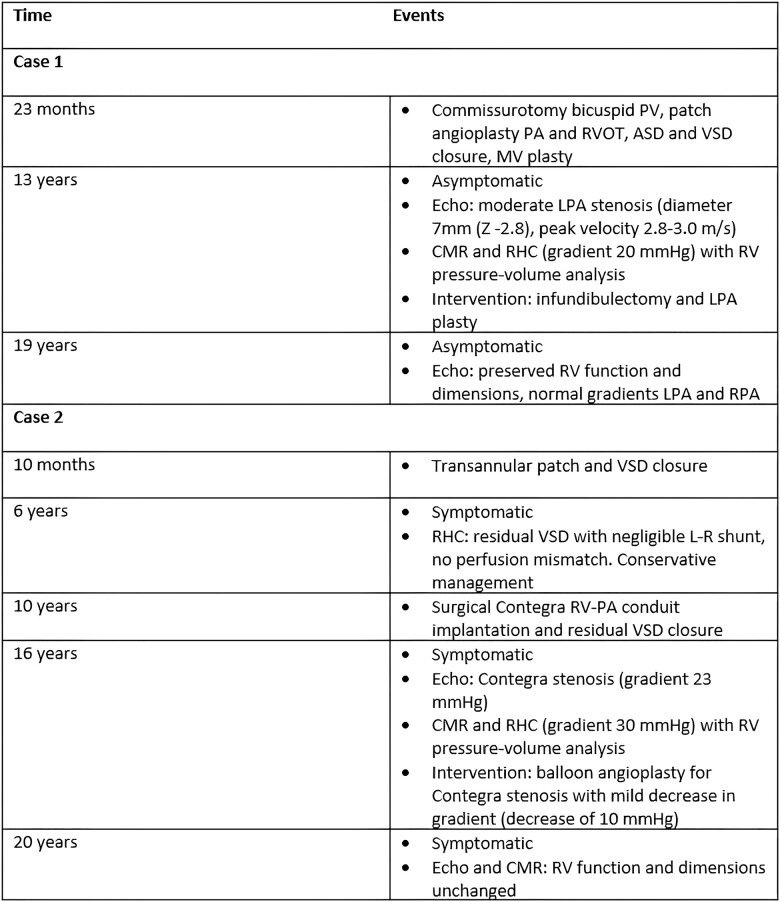
ASD, atrial septal defect; CMR, cardiac magnetic resonance; LPA, left pulmonary artery; MV, mitral valve; PA, pulmonary artery; PV, pulmonary valve; RHC, right heart catheterization; RPA, right pulmonary artery; RV, right ventricle; RV-PA conduit, right ventricular pulmonary arterial conduit; RVOT, right ventricular outflow tract; ToF, tetralogy of Fallot; VSD, ventricular septal defect.

## Case presentation

### Patient 1

**Figure 2:**
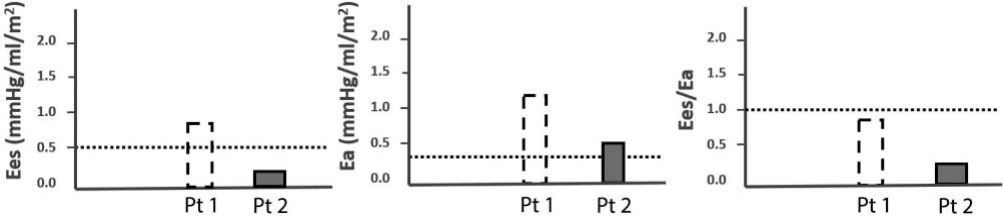
Right ventricular to pulmonary arterial coupling in two cases with tetralogy of Fallot and right ventricular pressure overload. Right ventricular to pulmonary arterial (RV-PA) coupling measurements in two cases with repaired ToF and RV pressure overload compared with reference values for RV-PA coupling from healthy literature controls. The dotted line represents the reference value for Ees, Ea, and Ees/Ea in healthy controls according to the literature.^13^ The end-sysolic elastance (Ees), which is considered a load-independent measure of ventricular contractility, was calculated as the slope of the end-systolic pressure–volume relationship (ESPVR) using (RV maximal isovolumetric pressure − RV systolic pressure)/stroke volume (Ees normal RV = ≥0.5, Ees Patient 1 = 0.9, Ees Patient 2 = 0.1).^7^ The maximal isovolumetric pressure of the RV was extrapolated from RV pressure curves. The Ea, which is mainly a reflection of pulmonary vascular resistance, was calculated as RV afterload using RV systolic pressure/stroke volume (Ea normal RV = ≤0.31, Ea Patient 1 = 1.2, Ea Patient 2 = 0.5).^9^ The ratio of Ees/Ea (RV-PA coupling) represents the efficiency of energy transfer from the RV to the pulmonary vasculature (Ees/Ea normal RV = ≥1, Ees/Ea Patient 1 = 0.8, Ees/Ea Patient 2 = 0.2).

**Figure 3:**
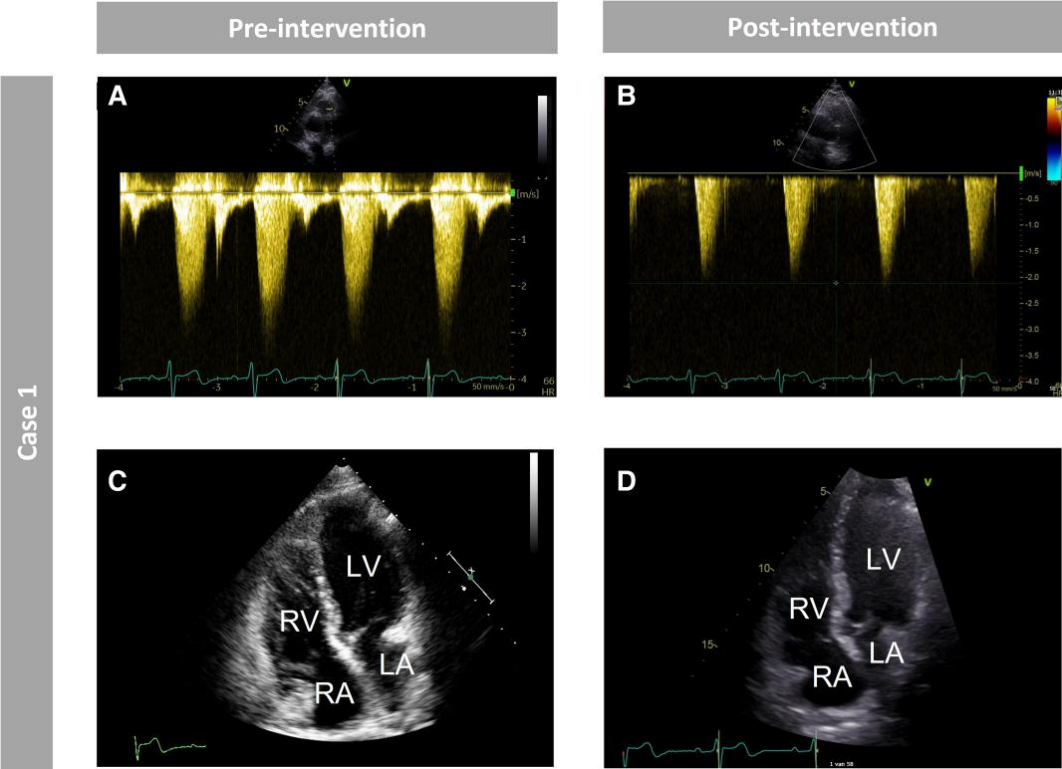
Pre- and post-interventional imaging of Case 1. *Figure 3* shows the pre- and post-interventional imaging of Case 1 using transthoracic echocardiography. (*A*) Pre-interventional peak gradient of the left pulmonary artery (LPA) measured using continuous wave Doppler echocardiography. (*B*) Post-interventional peak gradient of the LPA measured using continuous wave Doppler echocardiography. Note the different scales in the vertical axis in panels *A* and *B*. (*C*) Pre-interventional four-chamber cardiac magnetic resonance cine image. (*D*) Post-interventional RV focused apical four-chamber echocardiography image. *A*–*B* show a decrease in post-interventional LPA peak gradient compared with pre-interventional LPA peak gradient using continuous wave Doppler echocardiography. *C*–*D* show pre- and post-interventional preserved RV volumes. Right ventricular systolic function was found to be preserved during echocardiography.

In a patient with trisomy 21, ToF was repaired by a commissurotomy for a bicuspid pulmonary valve, a mean pulmonary artery, and right ventricular outflow tract (RVOT) patch, atrial septal defect and ventricular septal defect closure, and mitral valve plasty at the age of 23 months. At the age of 13, he was asymptomatic with no signs of venous congestion [physical examination: 144 cm, 43 kg, body mass index (BMI) 21, body surface area (BSA) 1.30, non-invasive blood pressure 128/62(84), New York Heart Association (NYHA) class I], but echocardiography showed a moderate left pulmonary artery (LPA) stenosis [diameter 7 mm (Z −2.8), peak velocity 2.8–3.0 m/s] and mild pulmonary valve regurgitation (peak gradient 2.3 m/s). Cardiac magnetic resonance demonstrated a perfusion mismatch with a four times higher perfusion to the right lung compared with the left lung. Biventricular volumes and function were preserved and RV mass was increased (*Table 1*). Right heart catheterization demonstrated a moderate LPA origin stenosis (invasive gradient LPA-PA 20 mmHg). In addition, a subvalvular RVOTO with an aneurysm was found (invasive gradient PA-RV 24 mmHg). The RPA showed a minimal stenosis (invasive gradient RPA-PA 10 mmHg). Right ventricular pressure was severely increased (RV systolic pressure 65 mmHg vs. LV systolic pressure 91 mmHg), but RV volumes and function were preserved (*Tables 1 and 2*). Right ventricular pressure–volume analysis showed increased pulmonary Ea and RV Ees, but RV-PA coupling was relatively preserved (*Table 2*, *Figure 2*). Furthermore, RV end-diastolic pressures and RV Eed (a measure of RV diastolic stiffness) were increased. Three months later, an infundibulectomy and LPA plasty were performed. The post-operative course was complicated by a superior caval vein (SCV) tear for which resuscitation and emergency reoperation were required. During 6 years of follow-up, the patient did not develop any symptoms and RV function remained preserved (*Table 1*, *Figure 3*).

**Table 1:** Pre- and post-interventional echo and cardiac magnetic resonance measurements

	Patient 1		Patient 2	
	Pre-intervention	Post-intervention	Pre-intervention	Post-intervention
Age (years)	13	18	16	22
Echocardiography				
Visual RV function	Good	Good	Moderate	Moderate
RV dilatation	No	No	Yes	Yes
RV hypertrophy	Yes	Yes	No	No
TAPSE (mm)	17	20	20.5	16
CMR				
LVEDV (mL/m^2^)	64	—	104	114
LVESV (mL/m^2^)	22	—	68	69
LVSV (mL/m^2^)	42	—	36	45
LV mass (g/m^2^)	41	—	39	—
LVEF (%)	66	—	35	40
LV peak GLS (%)	−25	—	−8	—
HR (beats/min)	76	—	83	72
CO (L/min)	3.2	—	3.0	3.2
RVEDV (mL/m^2^)	61	—	114	117
RVESV (mL/m^2^)	19	—	72	72
RVSV (mL/m^2^)	42	—	41	45
RV mass (g/m^2^)	27	—	19	—
RV relative wall thickness (g/mL)	0.44	—	0.17	—
RVEF (%)	68	—	37	39
RV FW peak GLS (%)	−29	—	−7	—

*Table 1* shows the pre- and post-interventional measurements using echocardiography and CMR in two cases with ToF and RV pressure overload. RV relative wall thickness was calculated as RV mass/RVEDV.^11^ LV GLS, left ventricular global longitudinal strain; RV FW GLS, right ventricular free wall global longitudinal strain.

**Table 2:** Right heart catheterization measurements including right ventricular to pulmonary arterial coupling parameters

	Patient 1	Patient 2	
	Rest	Rest	Adrenaline
RHC parameters pre-intervention			
LV Psys (mmHg)	91	98	140
RV Psys (mmHg)	65	31	65
RV Ped (mmHg)	13	7	—
MPA Psys (mmHg)	41	20	35
LPA Psys (mmHg)	21	15	—
RPA Psys (mmHg)	33	15	—
mPAP (mmHg)	21	—	—
PCWP (mmHg)	12	—	—
RV wall tension (mmHg)	127	324	NA
RV wall tension (kPa)	17	43	NA
PVR (wood units per m^2^)	2.8	—	NA
RV compliance	1.0	2.8	NA
RHC parameters post-intervention			
LV Psys (mmHg)	NA	—	135
RV Psys (mmHg)	NA	—	55
MPA Psys (mmHg)	NA	—	35
RV-PA coupling parameters			
Ees	0.9	0.1	—
Ea	1.2	0.5	—
Ees/Ea	0.8	0.2	—
Eed (measure of RV diastolic stiffness)	0.9	0.2	—

*Table 2* shows the measurements during right heart catheterization (RHC) including the RV-PA coupling parameters. Right ventricular to pulmonary arterial coupling parameters were only assessed before the intervention. End-sysolic elastance, which is considered a load-independent measure of ventricular contractility, was calculated as the slope of the end-systolic pressure–volume relationship (ESPVR) using (RV maximal isovolumetric pressure − RV systolic pressure)/stroke volume.^7^ The maximal isovolumetric pressure of the RV was extrapolated from RV pressure curves. Ea, which is mainly a reflection of pulmonary vascular resistance, was calculated as RV afterload using RV systolic pressure/stroke volume.^9^ Right ventricular diastolic stiffness was assessed using RV end-diastolic elastance (Eed). End-diastolic elastance was calculated as the slope of the end-diastolic pressure–volume relationship (EDPVR) at end-diastole.^10^ Right ventricular wall tension was calculated according to Laplace’s law = 0.5 × RV systolic pressure × RV end-systolic radius divided by RV end-systolic wall thickness.^12^

Ea, arterial elastance; Eed, end-diastolic elastance; Ees, end-systolic elastance; Ees/Ea, right ventricular to pulmonary arterial coupling; NA, not applicable; PCWP, pulmonary capillary wedge pressure; Ped, end-diastolic pressure; Psys, systolic pressure; PVR, pulmonary vascular resistance.

### Patient 2

**Figure 4:**
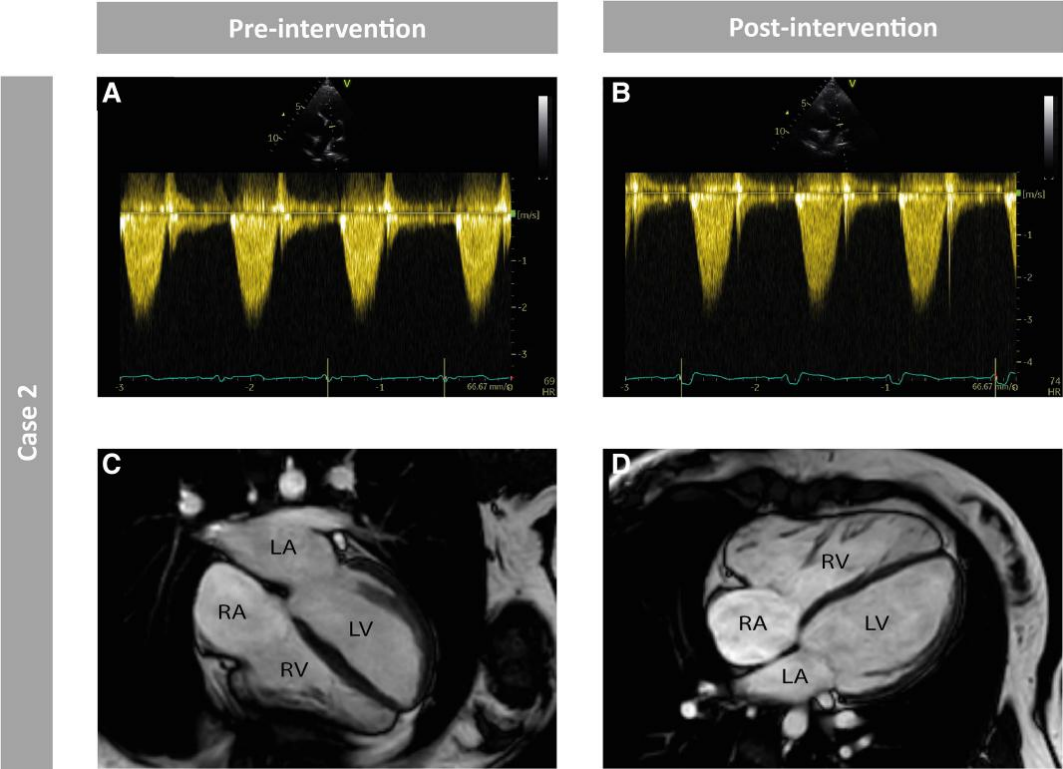
Pre- and post-interventional imaging of Case 2. *Figure 4* shows the pre- and post-interventional imaging of Case 2 using transthoracic echocardiography and cardiac magnetic resonance imaging. (*A*) Pre-interventional peak gradient of the right ventricular outflow tract (RVOT) measured using continuous wave Doppler echocardiography. (*B*) Post-interventional peak gradient of the RVOT measured using continuous wave Doppler echocardiography. Note the different scales in the vertical axis in panels *A* and *B*. (*C*) Pre-interventional four-chamber cardiac magnetic resonance (CMR) cine image. (*D*) Pre-interventional four-chamber CMR cine image. *A*–*B* show no decrease in post-interventional RVOT peak gradient compared with pre-interventional RVOT peak gradient using continuous wave Doppler echocardiography. *C*–*D* show pre- and post-interventional RV dilatation. Right ventricular systolic function was found to be reduced during CMR.

A female patient with ToF was corrected with a transannular patch and a ventricular septal defect (VSD) closure at the age of 10 months. At the age of 6 years, she was symptomatic. She suffered from complains of fatigue and showed reduced exercise capacity and shortness of breath when walking the stairs during daily life. A residual VSD with negligible L-R shunt was found on echocardiography. Since there was no perfusion mismatch, management was conservative. Three years later, she remained symptomatic. Physical examination was unremarkable. Cardiopulmonary exercise testing showed reduced exercise capacity (VO2 max/kg: 38 mL/kg/min, Z −0.4), and CMR demonstrated left ventricular (LV) and RV dilatation and systolic impairment (*Table 1*). A surgical closure of the residual VSD was performed, and a valved RV-PA conduit (Contegra 18 mm) was implanted. However, exercise capacity remained unchanged. Six years later, she was still symptomatic with no signs of venous congestion and had impaired exercise capacity (physical examination: 169 cm, 54 kg, BMI 19, BSA 1.61, non-invasive blood pressure 113/66(82), NYHA class II). Echocardiography revealed a stenosis of the Contegra RV-PA conduit (estimated echocardiographic gradient 23 mmHg) and mild PR. Right heart catheterization showed the following invasive pressures and gradients at rest: LV systolic pressure 98 mmHg, RV systolic pressure 31 mmHg, mean arterial pressure (MPA) systolic pressure 20 mmHg, and LPA/RPA systolic pressure 15 mmHg. A minimal LPA and RPA stenosis (invasive gradient both 5 mmHg) and a trivial Contegra stenosis (invasive gradient 11 mmHg) were found. Because of hypotension under general anaesthesia, an adrenaline challenge was performed (LV systolic pressure 140 mmHg, RV systolic pressure 65 mmHg, and MPA systolic pressure 35 mmHg). This revealed a moderate Contegra stenosis (invasive gradient 30 mmHg). Right ventricular pressure was mildly increased (rest: RV systolic pressure 31 mmHg vs. LV systolic pressure 98 mmHg, adrenaline: RV systolic pressure 65 mmHg vs. LV systolic pressure 140 mmHg). However, there was severe RV dilatation and RV systolic dysfunction (*Tables 1 and 2*). Right ventricular pressure–volume analysis showed that Ea was increased but Ees was severely reduced leading to severe RV-PA uncoupling. Furthermore, RV end-diastolic pressures and RV Eed were preserved (*Table 2*, *Figure 2*). The Contegra stenosis was corrected using balloon angioplasty because of the lack of an ideal landing zone for stenting. After the balloon angioplasty, there was a mild decrease in the Contegra gradient (invasive gradient pre-intervention 30 mmHg vs. 20 mmHg post-intervention). No pulmonary valve regurgitation was present after balloon angioplasty (echocardiographic PV gradient 32 mmHg, Vmax 2.8 m/s). Despite this intervention, exercise capacity remained impaired during follow-up. Right heart catheterization revealed a good Contegra function and unchanged mild LPA stenosis 2 years after Contegra balloon angioplasty. However, CMR revealed persistent LV dysfunction (LVEF 40%) and unchanged RV function (RVEF 39%) 4 years after Contegra balloon angioplasty (*Table 1*, *Figure 4*).

## Discussion

Our case series of two patients with repaired TOF and a supravalvular RVOTO shows that, despite high RV pressures, RV function may be maintained after RVOTO intervention and during long-term follow-up. Of interest, we demonstrated that Ea and RV Ees were increased in Patient 1 and RV-PA coupling remained relatively preserved using RV pressure–volume analysis. In contrast, we demonstrated in Patient 2 that despite a mild increase in RV pressures, RV function may deteriorate and the RV may be uncoupled from the pulmonary arterial load. Relief of the RVOTO did not result in reversal of RV dysfunction.

Our case series is the first that performed single-beat RV pressure–volume analysis in patients with repaired ToF. We present two cases with a supravalvular RVOTO but a different response of the RV to the elevated afterload. This is in line with the study from Latus et al.^5^ who found no relationship between RVOT gradients and RV function. We found that both patients had increased RV pressures due to RVOTO. However, in Patient 1, RV pressures and Ea were more than 1.5-fold higher compared with Patient 2. Of interest, RV Ees was increased in Patient 1, which is in line with previous literature.^14^ The increase in RV contractility (Ees) might be achieved by the increase in wall thickness as well as changes in muscle properties. Similar findings of a supranormal Ees and hypercontractile RV have been found in patients with pulmonary hypertension (PH).^15^ Despite high RV pressures, Patient 1 showed preserved RV volumes and systolic function and a relatively maintained RV-PA coupling. Of interest, RV function remained preserved during long-term follow-up. In contrast, in Patient 2, we found that despite mildly increased RV pressures, significant RV-PA uncoupling occurred, with no to little improvement in RV function after RVOTO intervention. This is in line with literature showing that RV function may be unchanged after surgical pulmonary valve replacement and underlines the importance of early detection of RV dysfunction.^16^ In addition, severe RV dilatation, low relative wall thickness, and increased RV wall stress were observed. Our findings are in line with previous studies which estimated RV-PA coupling non-invasively or after the application of inotropes in patients with repaired ToF.^17,18^ However, these studies were performed in repaired ToF patients with mainly residual volume overload. In addition to increased RV pressure and volume overload, RV-PA uncoupling might be explained by different disease stages, gender, age and differences in (the history of) PR.^18,19^ Therefore, a complex combination of factors might have impact on RV-PA coupling in repaired ToF. Of interest, Patient 1 showed increased RV end-diastolic pressures and RV diastolic stiffness. This might be a consequence of stiffening of the cardiomyocytes due to compensatory RV hypertrophy in response to pressure overload. Right ventricular hypertrophy might protect against progressive RV dilatation and reduces ventricular wall stress.^20^

### Clinical implications

Right ventricular function determines long-term outcome in patients with repaired ToF, and therefore, preservation is of great importance. However, timing of re-interventions for the relief of RVOTO remains challenging. In line with previous literature, our case series shows that RV pressures do not always inform us properly about the extent of RV adaptation and RV function.^5,20^ In addition, this case series is the first to perform single-beat RV pressure–volume analysis in patients with ToF and provides new insights in this extra tool that might help to optimize the timing of re-interventions. We found that linking indexes of RV function to its afterload, in addition to RVOT gradients as recommended by the guidelines, might be of additional value for risk stratification, monitoring, and optimizing timing of re-interventions in patients with repaired ToF.^3,4^

### Limitations

In this case series, we present two patients with differences in age, gender, and surgical history, which might have impact on our results. Right ventricular pressure–volume analysis was performed using the single-beat method from Brimioulle et al.^7^ However, a disadvantage of this method is that it is based on a few assumptions.^7^ Despite these assumptions, the single-beat RV pressure–volume analysis is a good and applicable alternative in patients for the multi-beat RV pressure–volume analysis. This multi-beat technique requires preload alterations using (partial) vena cava occlusion or the Valsalva manoeuver, which might not be feasible in all circumstances. In addition, Ea was used to determine RV afterload. However, Ea mainly represents pulmonary vascular resistance, which was only mildly increased in this case series. Therefore, other factors such as pulmonary arterial compliance might also have an impact on RV afterload. Finally, we solely report on single time point RV pressure–volume analysis. Future studies with multiple time point RV pressure–volume analysis would be of great interest. This supports future larger and prospective studies about RV pressure–volume analysis in repaired ToF.

## Conclusions

This case series illustrates that development of RV dysfunction might not be predicted by the severity of RVOTO in patients with repaired ToF. Linking indexes of RV function to its afterload might be of additional value for risk stratification, monitoring, and optimizing timing of re-interventions in patients with repaired ToF

## Data Availability

The data underlying this article will be shared upon reasonable request to the corresponding author.

## Lead author biography

Renée S. Joosen is a PhD candidate at the Department of Pediatric Cardiology at the University Medical Center Utrecht, in Utrecht, the Netherlands. Her areas of interest include congenital heart disease, right ventricular function, and cardiac imaging

**Consent:** The author(s) confirm that written consent for submission and publication of this case report has been obtained from the patients in line with COPE and ICMJE recommendations.

**Funding:** We acknowledge the support from the Netherlands Cardiovascular Research Initiative to this case series as funding source for R.S.J.’s PhD project as part of the OUTREACH consortium: an initiative with support of the Dutch Heart Foundation and Hartekind, CVON2019-002 OUTREACH.
